# Aeromedical Transport of Critically Ill Infants Less Than 3 Months of Age

**DOI:** 10.1177/2333794X17739743

**Published:** 2017-11-14

**Authors:** Anil P. George, Akshay Sharma, Scottie B. Day

**Affiliations:** 1 University of Illinois College of Medicine, Department of Pediatric Hematology/Oncology, Chicago, IL, USA; 2 St. Jude Children’s Research Hospital, Memphis, TN, USA; 3 University of Kentucky, Department of Pediatric Critical Care, Lexington, KY, USA

Aeromedical transport is a modality for emergently transporting critically ill infants to facilities with pediatric intensive care units and access to pediatric subspecialists. Current commercial air travel guidelines recommend against aeromedical transport of infants less than 1 week of age as well as high-altitude travel (defined as more than 2500 meters above mean sea level or approximately 1.5 miles) for healthy infants less than 6 weeks of age.^1-3^ Further in situ flight research and observational studies reveal that there is an increased risk of hypoxemia in preterm infants and in those with respiratory disease, but not in healthy full-term infants who are older than 6 months of age.^1,3-6^ Aeromedical transport potentially exposes infants to altitude hypoxia, making them susceptible to hypoxemia.^3-5^ There is a need to learn more about the effects of altitude hypoxia on transporting critically ill neonates as many of these patients require transport to facilities with access to pediatric subspecialists for ongoing medical care. We present findings of a retrospective, observational pilot study examining the effects of altitude hypoxia during aeromedical transport of critically ill infants younger than 3 months of age.

Prior to engaging in this study, approval was obtained from the University of Kentucky Institutional Review Board. From January 2013 through December 2014, 108 infants less than 3 months of age were transported using a rotary-wing aircraft (helicopter) by the transport team of the University of Kentucky Children’s Hospital. The transport team comprised a nurse-nurse team. Infants requiring supplemental oxygen prior to transport, having evidence of carbon monoxide exposure, and having evidence of temperature instability were excluded from the study. A total of 41 infants met the criteria to be included for analysis. Continuous pulse oximetry using the Nellcor Pulse Oximeter was performed during the entire flight duration and the lowest serum oxygen serum blood oxygen saturation (SpO_2_) recorded was used to calculate the index of desaturation (index of desaturation defined as SpO_2_ less than 94%). On the day of transport, the median age of subjects was 0.7 days (range = 0-80 days), median postmenstrual age was 37.6 weeks (range = 33.7-51.4 weeks), median flight duration was 85 minutes (range = 5-176 minutes), median flight altitude was 3000 to 3500 feet by Visual Flight Rules and 4000 feet by Instrument Flight Rules, and median distance flown was 100.2 nautical miles (range = 24-178.8 nautical miles).

We observed that the SpO_2_ fell below 94% in 4 infants (Table 1) in our patient cohort, of whom 3 patients had congenital cyanotic heart diseases (1 patient with transposition of the great arteries, 2 patients with hypoplastic left heart syndrome) and hence had lower oxygen saturation due to mixing of oxygenated and deoxygenated blood. No adverse events (defined as, apnea, hypothermia, hemodynamic instability, or death) were observed in any of these infants and only 5 infants required any supplemental oxygen support via nasal cannula or oxyhood during transport.

**Table 1.** In-Flight Oxygen Saturation Levels According to Postmenstrual Age.

Infant Age	No. of Infants	No. of Infants With Desaturation SpO_2_ <94%, n (%)	Lowest Median In-Flight SpO_2_
Postmenstrual age <37 weeks	16	0 (0%)	97 (95-100)
Postmenstrual age >37 weeks and Postnatal age ≤1 week	20	2 (10%)	98 (77-100)
Postnatal age >1 week to 6 weeks	3	1 (33.3%)	94 (85-97)
Postnatal age >6 weeks to 3 months	2	1 (50%)	91 (83-100)
Total	41	4 (9.8%)	97 (77-100)

The factors that contribute to altitude hypoxia in infants include mismatch between ventilation and perfusion (V/Q mismatch), the presence of fetal hemoglobin, pulmonary vasoconstriction and bronchoconstriction in the presence of airway hypoxia, and smaller airway diameter.^4-6^ The American Academy of Pediatrics Guidelines for Air and Ground Transportation of Pediatric Patients state that the most important determinant of transportation modality should be guided by the optimal interhospital transport time based on the patient’s clinical condition.^7^ The advantages of helicopter transport include rapid transport time for relatively short distances and the ability to reach otherwise inaccessible areas.^7^ The British Thoracic Society 2004 Guidelines for commercial air travel suggest that infants with a recorded SpO_2_ below 90% receive supplemental oxygen.^2^ These guidelines, however, are not evidence-based recommendations and do not provide direction for rotary-wing transport of ill infants.^1,3,5^

Our study was limited by a small sample size, no calculable power, and the lack of prospective data collection. However, our study is one of the first to analyze the safety of the rotary-wing transport of neonates. Additionally, our patient cohort did not have any adverse events. Based on these preliminary findings, we would not recommend delaying aeromedical transport of sick infants less than 3 months of age. In our specific study, the University of Kentucky Children’s Hospital encompasses a large catchment area that extends from Central Kentucky to West Virginia and Virginia, states that border the eastern border of Kentucky. Our institution serves as one of the only tertiary care centers and medical homes in this area with pediatric subspecialists. Similar logistical challenges are also present at several pediatric academic institutions across the United States. Potentially exposing critically ill infants to altitude hypoxia must be balanced against the benefit of transporting them quickly to an intensive care unit for continued medical management. Future directions for investigation include prospective, multicenter analyses; developing guidelines for the aeromedical transport of patient cohorts without established guidelines including patients with congenital heart disease and patients with venous thromboembolism; assessing ambient influences during aeromedical transport and its effect on oxygenation; and analyzing the risk of aeromedical transport of infants with parenchymal lung disease.^3^

## Author Contributions

APG: Contributed to conception or design; contributed to acquisition, analysis, or interpretation; drafted the manuscript; critically revised the manuscript; gave final approval; agrees to be accountable for all aspects of work ensuring integrity and accuracy.

AS: Contributed to conception or design; contributed to acquisition, analysis, or interpretation; critically revised the manuscript; gave final approval; agrees to be accountable for all aspects of work ensuring integrity and accuracy.

SBD: Contributed to conception or design; critically revised the manuscript; gave final approval; agrees to be accountable for all aspects of work ensuring integrity and accuracy.
